# Behavioral models in psychopathology: epistemic and semantic considerations

**DOI:** 10.1186/s12993-019-0152-4

**Published:** 2019-03-01

**Authors:** Caio Maximino, Franz Josef van der Staay

**Affiliations:** 1 Laboratório de Neurociências e Comportamento, Grupo de Pesquisas em Neurofarmacologia e Psicopatologia Experimental, Instituto de Estudos em Saúde e Biológicas, Universidade Federal do Sul e Sudeste do Pará, Unidade III - Av. dos Ipês, S/N, Marabá, Brazil; 2 Behavior and Welfare Group, Department of Farm Animal Health, Veterinary Faculty, University Utrecht, P.O.Box 80.151, 3508 TD Utrecht, The Netherlands

**Keywords:** Animal model, Behavioral phenotyping, Experimental psychopathology, Validity

## Abstract

The use of animals in neurosciences has a long history. It is considered indispensable in areas in which “translational” research is deemed invaluable, such as behavioral pharmacology and comparative psychology. Animal models are being used in pharmacology and genetics to screen for treatment targets, and in the field of experimental psychopathology to understand the neurobehavioral underpinnings of a disorder and of its putative treatment. The centrality of behavioral models betrays the complexity of the epistemic and semantic considerations which are needed to understand what a model is. In this review, such considerations are made, and the breadth of model building and evaluation approaches is extended to include theoretical considerations on the etiology of mental disorders. This expansion is expected to help improve the validity of behavioral models and to increase their translational value. Moreover, the role of theory in improving construct validity creates the need for behavioral scientists to fully engage this process.

Models are of central importance in many different scientific contexts: in physics, different models of the atom substituted each other, and are central to understand how atomic and subatomic particles behave; the double helix model of DNA, the Lotka–Volterra model of predator–prey interaction, agent-based models in economics, and the Rescorla–Wagner model of classical conditioning are all central in their respective domains. Indeed, scientists spend so much time building, testing, comparing, and revising models that these tools can be considered one of the principal instruments of contemporary science [28, 29].

The current perspective in behavioral neuroscience is characterized by an international trend to appeal to “translational” perspectives in the health sciences: that is, research which aims to “‘translate’ understanding into effective strategies to control organisms, processes, or events” [9, p. 1568]. This, of course, is the setting stage for every approach to behavioral modeling. Behavioral models (a subset of animal models) are the main tool in contemporary experimental psychopathology, and much of our understanding on the psychological and biological mechanisms underlying mental disorders arises from studies using such tools. What, however, is a model in experimental psychopathology? In what sense does it *represent* a human disorder? What are animal models? How do we learn from models? In an attempt to answer these questions the present article summarizes current semantic and epistemological questions regarding behavioral models, and attempts to extend current theories of validity by framing them under the reference of the diathesis–stress theory.^1^

## Behavioral models as analogies

While many definitions of a animal model exist, a synthesis has been proposed by van der Staay [70] as follows:

An animal model with biological and/or clinical relevance in the behavioral neurosciences is a living organism used to study brain–behavior relations under controlled conditions, with the final goal to gain insight into, and to enable predictions about, these relations in humans and/or a species other than the one studied, or in the same species under conditions different from those under which the study was performed (pp. 133–134).

Behavioral models maintain a relationship of analogy with the modeled disorder; that is, a given behavioral model represents a psychopathology. As material analogies, behavioral models are a comparison between the model and its target that is defined in terms of their properties and the relationships between them [2]. Horizontal relationships in an analogy are those of similarity and dissimilarity between the properties of the model and its target, while vertical relationships are the causal relationships that hold between the properties of the model and its target [37]. If two analogues can be shown to share identical or very similar sets of horizontal relationships, then whatever is known to be a causal relationship between those properties in the model can be assumed to be present in the target as well [37].

The representational nature of an animal model (i.e., its semantic dimension) is also of consequence to its epistemic dimension—that is, whether or not a model is useful to produce new knowledge regarding its target disorder depends on what it means to say that the model represents the disorder. This epistemic dimension delimits the translational relevance of the model: animal modeling can be understood as a process of extrapolation [60], “where researchers first establish the biological mechanisms that are at work in animals and then use this information to infer what might be happening in humans (perhaps with the secondary goal of establishing more general biological principles)” [52, p. 6].

Exactly how this representation happens is subject to much controversy. For example, LaFollette and Shanks [46] argued that all animal models (including behavioral models) are either causal analog models (CAMs) or hypothetical analog models (HAMs). For a model to be a CAM of a human phenomenon (e.g., a given mental disorder), both must share certain properties such that if a novel property is observed in the model it is likely that this property is also found in humans; for this to happen, the novel property must be causally linked to the previously identified properties. Moreover, for a model to be a CAM, the requirement of absence of differences which are causally relevant for the disease being modeled, especially in terms of etiology (“intervention disanalogies”) or evolutionary history (“intrinsic disanalogies”)—the “causally relevant disanalogies” in the terminology of LaFollette and Shanks [46]—between the target disorder and the model must be added [20]. This is a strong requirement for vertical relationships: in order for a causal relationship to be established, not only horizontal relationships must hold, *but causally relevant disanalogies must not be present.* Sjoberg [63] argued that, while researchers should be cautious in using arguments from analogy, and it is highly unlikely that any single model reach the criteria for being a CAM, “the strength of an animal model is to generate new knowledge and hypotheses relevant to the target group, including the assessment of potentially useful treatments, but that these new possibilities are only hypothetical once they are discovered” (p. 9).

This latter definition is what LaFollette and Shanks [46] call a HAM—that is, the HAM is an heuristic device that has enough similarities with its target that it can be used to generate novel hypotheses, but not to establish causal relationships. HAMs have properties which are functionally similar to some properties of the target; it does not follow that model and target are causally similar—that is, the existence of horizontal relationships does not entail the existence of vertical relationships. This fallacy, termed “the modeller’s functional fallacy” by the authors, fails to acknowledge that model and target differ in many crucial respects (that is, there are causally relevant disanalogies) and therefore results from animal models cannot necessarily be transposed to humans. Put in another way, a HAM is not a CAM, but neuroscientists frequently treat it otherwise. Sjoberg [63] cautions against arguing from analogy, stating that researchers should recognize the limitations of animal models. This critique echoes important arguments against the use of animal models in psychology [62]. From this critique it follows that animal models are not ideal tools for direct testing and extrapolation, but rather they are “heuristic devices”—sources of hypotheses with which to study human biological function and pathology [46].

Degeling and Johnson [20], however, argued that this position is an oversimplification of the way scientists understand the role of animal models. Indeed, they argued that a more extensive taxonomy of animal models is necessary to fully understand how scientists use these devices, and the CAM/HAM distinction is not enough:

“There are other epistemic nuances and distinctions relevant to the use of animal models in medical science, particularly in the context of trying to simultaneously describe and understand the epistemological validity of the practice, which are missed by the introduction of such a stark partition between just two types of models” [20, p. 96]

It is useful to expand the LaFollette-Shanks theory in reference to behavioral models. Hau [36] proposed that models are divided by two overlapping systems: first, by its purpose; and second by the relative similarity of the model to its target. From the point of view of purpose, models can be classified as exploratory, explanatory, or predictive. Exploratory models are used to investigate and manipulate biological mechanisms in order to generated hypotheses that can be tested (that is, are HAMs by definition).

“Observations derived from this type of modeling practice can inform the generation of hypotheses such that experimentation can continue along reductive lines to broaden scientific knowledge or the data produced can be tested and correlated against other model systems to refine the description and verify the uniformity and theoretical coherence of a different category of model, namely, an explanatory one” [20, p. 100].

Explanatory models are an epistemological refinement of exploratory models, and are integrated within a theoretical framework that has either isolated a specific mechanism or is judged to reliably represent the complexity of interacting mechanisms. Explanatory models are further refined, after producing reliable and reproducible data, to become a predictive model [36]—that is, it is subjected to quantified or qualified interference or disruption of function. Thus, the process of model building usually drives models from exploratory to explanatory to predictive models; in Baird’s [5] terms, exploratory models perform epistemic work as instruments that create or represent phenomena; explanatory models perform epistemic work as instruments that represent phenomena; and predictive models perform epistemic work as instruments that create phenomena and then allow their measurement to assess or predict analogous events in other organisms or systems. It is easy to understand that both explanatory and predictive models are further refinements of what LaFollette and Shanks [46] called a CAM.

While both LaFollette and Shanks [46] and Hau [36] proposed taxonomies of models that are based on epistemic work, Degeling and Johnson [20] proposed a taxonomy that is based on a specific dimension of epistemic work, that of *similarity*. Similarity can be understood in terms of fidelity (that is, the relative similarity of mechanism) and discriminating ability (the relative similarity of response to disturbances). From the point of view of similarity, models can be classified as homologous, isomorphic, or partial models [36]. Homology refers to the degree with which the mechanisms and their interactions and psychobiological consequences are identical in the model and its target; “within biomedical research, homologous models are those in which the etiology, symptoms, and outcome of the animal model duplicates those of the human disorder” [20, p. 101]. Thus, homologous models have high fidelity and discriminating ability.

Isomorphic models are those in which the mechanisms in the model and its target are identical but causally unrelated, and therefore have high discriminating ability but low fidelity. For example, in the amphetamine psychosis model of schizophrenia, neurochemical and behavioral alterations are similar to what is observed in schizophrenic patients [64], but these neurobehavioral changes are produced artificially in the laboratory in a way that does not reflect the human etiology. Therefore, isomorphic models have limited predictive capacities. Partial models have low fidelity and low discriminating ability, and “are poorly predictive but allow some isolated aspect of a more complex biological mechanism to be mapped and manipulated to generate further hypotheses of relevance to understanding” the target disorder [20, p. 101].

A classical taxonomy of models, proposed by Paul Willner [78, 79], also relies on concepts of similarity and purpose. Willner discriminated between screening tests (used to predict a desired drug activity), behavioral bioassays (used to study the physiological and neurobiological mechanisms that are associated with brain function), and simulations (which generally attempt to model mental disorders based on comparative studies of the same states and conditions). Screening tests are partial models not in the sense that they are not able to discriminate between predicted drug effects (for example, the tail suspension test can correctly discriminate between drugs that target the serotonin transporter and drugs that do not), but because results obtained from it are very limited in understanding the target disorder; in that sense, results from the tail suspension test cannot, in spite of what appears in the literature, be used to make inferences on depression. In the pharmaceutical industry screening tests are often the first step in in vivo drug target identification and selection of compounds for further drug development. Behavioral bioassays are isomorphic models in the sense that inferences made on the mechanisms of a specific brain function cannot necessarily be extrapolated to a disorder; for example, the elevated plus-maze is commonly used to understand non-pathological anxiety, and its use in inferring mechanisms of pathological anxiety is limited. The purpose of behavioral bioassays is to develop hypotheses about normal function. Simulations involve attempts to model the etiology, symptoms, and outcome of the human disorder, and are homologous. The epistemic work made by simulations is the creation of a phenomenon (a diseased organism) that is used to assess or predict analogous events in humans with mental disorders.

This distinction stresses the idea that only simulations are true models, and screening tests and behavioral bioassays are actually tests. Simulations are more commonly used to study the pathophysiology and treatment of mental disorders, but they are also useful to produce insights into normal function [17]. Simulations should require the use of animals with pathological organisms, and therefore are highly dependent on the criteria and restrictions of validity. While some behavioral bioassays use pathological organisms (for example, the olfactory bulbectomy test, in which ablation of the olfactory bulb produces learning deficits, hyper-reactivity, and glucocorticoid responses which are reversed by antidepressants [15]; or the stress-induced hyperthermia, in which transferring rats or mice to a novel environment increases body temperature in a anxiolytic drug-sensitive way [53], it is not *required* of them that the induction methods are analogs of etiological factors of the target disorder. Similar observations can be made for most research on transgenic organisms (e.g., knockout rodents).

Strictly speaking, animal models should be hypothesis-based [63, 71]. As we discuss below (“Models verus tests” section), one important difference between tests and “true” models (simulations, sensu Willner, or homologous models) is the focus of the latter on construct validity; as a consequence, a true model must be grounded on theory, and therefore be hypothesis-driven. The focus of the last generation on high-throughput tests to detect the effects of gene mutations [67] produced hypothesis-independent “exploratory models” sensu Hau. It has repeatedly been stated that high-throughput behavioral assays are *sine qua non* conditions of appropriate screening procedures, especially in the pharmaceutical industry [12, 30, 67]. “But if the goal of high-throughput screens is to achieve understanding of gene function or behavior, these efforts may be misguided” [17, p. 1177], because while throughput increases assay sensitivity and specificity, sometimes resulting in better predictive validity, a behavioral bioassay or a simulation focus extensively on other aspects of predictive validity (e.g., induction validity) and on construct validity. We propose, therefore, a refinement of Hau’s [36] scheme by suggesting that only explanatory and predictive models are “true models”, while exploratory models are in fact tests (see “Models versus tests” section, below, for a distinction between models and tests). Likewise, only homologous models/simulations should be considered “true models”.

While fidelity and similarity are both relevant to the epistemic veracity of a behavioral model, the focus of researchers in the field is increasingly shifting towards the sensitivity of a model to disturbances (discriminating ability) [20]. For example, it has been suggested that while using normal animals (i.e., animals without any observable behavioral deficit) is useful for basic pharmacological assays (i.e., screening tests) or to investigate the neurobiology of normal brain function (i.e., biobehavioral assays), the characterization of a behavioral model (i.e., simulation) necessitates the use of animals with naturally occurring or experimentally induced deficits [70].

The relationship between all these definitions is not straightforward, because some focus on purpose, some on definitions of validity, and some on epistemic work. Table 1 attempts to clarify the issue by summarizing the definitions. We chose to base the summary on Willner’s taxonomy due to its profound influence on behavioral researchers, while the other taxonomies are more representative in philosophy of science. As shown in the Table, strictly speaking, *the argument from analogy means that only simulations can be treated as models*. Moreover, the Table also underlines the oversimplification of using the LaFollette and Shanks [46] taxonomy, as even simulations cannot always be understood as CAMs, but provide more important epistemic work that cannot be understood by the simplistic concept of HAM.

**Table 1:** Distinction between types of tests and models, with a focus on their purpose and epistemic work

Category	Type	Purpose	Epistemic work
Tests	Screening tests	Allows limited comprehension of an isolated aspect of a complex biological mechanism to be mapped Limited predictive usefulness (e.g., predicting desired drug activity)	Low fidelity (underlying mechanism does not need to be similar) Low discriminating ability (not necessarily sensitive to, e.g., triggering factors) Not necessarily hypothesis-driven Low construct validity; moderate predictive validity (pharmacological isomorphism only)
	Biobehavioral assays	Allows broader comprehension of a mechanism, without necessary causal analogy Moderate predictive usefulness [e.g., studying neural bases of behavioral (dys)functions]	Low fidelity (mechanism similar, but not causally analogous) High discriminating ability (sensitive to disturbances by definition) High predictive validity, at best moderate construct validity
Models	Simulations	Can allow inferences and extrapolation to the human disorder, with high probability that the hypothesis thus generated is true	High fidelity (similar mechanisms with probable causal analogy) High discriminating ability (sensitive to disturbances by definition) High face, predictive, and construct validity (considers the need to address theoretical constructs on the etiology, symptomatology, and treatment)

## Models versus tests

The idea that a behavioral model is an analogy is interesting, but the terms of the analogy are not clear from the beginning. The target is usually a disorder, but model building is a reductive task in the sense that certain variables are valued and identified as relevant in detriment to others; models are usually less complicated than the thing being modeled [35]. As a consequence, what is modeled is not the entire disorder, but rather one or a few aspects of it.

Some consider the “classical” definition of a model—an apparatus plus an animal, along with instructions on how to make both interact to produce meaningful behavior—to be restrictive, and instead consider these endpoints to be tests for a given behavioral domain which may or may not be altered in a disorder. This is the approach we took on Table 1. Geyer and Markou [31] argued that while testing therapeutic manipulations under “baseline” conditions—i.e., without an explicit inducing manipulation—can have face validity and pharmacological isomorphism, it lacks most aspects of predictive and construct validity. From the pharmacological point of view, “the mechanisms through which drugs produce their effects in ‘normal’ versus perturbed animals may differ, even if the primary neurochemical effect may be the same” [31, p. 449].

In that sense, the rat elevated plus-maze, the rat Porsolt forced swim test, and the zebrafish light/dark test are not models per se, but rather tests for anxiety and behavioral despair. Van der Staay [70] argued that these endpoints represent models only when they are dependent variables in an experimental approach in which the independent variable is a ‘model animal’, of which there are two kinds: normal animals (the “baseline” alluded by Geyer and Markou [31]), and animals with deficits. These deficits can be naturally occurring (including aging; spontaneously and endogenously occurring behavioral or neurological alterations; spontaneously occurring mutations; genetic lines; and selected extremes from a particular population) or experimentally induced (including transgenic and knockout animals; animals from mutagenesis screens; selection lines resulting from breeding for a particular endophenotype; animals with electrical or pharmacological disruptions; animals with CNS-specific lesions; animals with altered developmental trajectories [e.g., postnatal stress]; and animals with cerebral ischemic or hemorrhagic stroke). Consequently, the process of modeling is the application of a set of independent variables which, in accordance to the best theories about the target disorder in humans, is expected to produce a neurobehavioral phenotype—that is, an effect on a set of dependent variables, the test.

The idea that only some aspects of the disorder need to be modeled is meant to answer to common criticisms regarding the impossibility of modeling a mental disorder due to the need to rely on verbal reports to infer some symptoms; so, for example, while it is not possible to infer whether an animal presents “fear of losing control or going crazy” after a situation which would trigger a panic attack, one can observe behavioral (avoidance, escape attempts, exophtalmia, etc.) and physiological effects (altered breathing, increased heart rate). There is, however, no consensus on exactly what aspect of the disorder is modeled. One common reductive approach in the current *zeitgeist* is the decomposition of complex mental disorders into endophenotypes, simpler neurobiological and physiological components which (being genetically determined and evolutionary conserved) “optimize” reductionism [6, 33, 34, 54]. An endophenotype carries certain characteristics which favor its choice; not every genetically determined, evolutionarily conserved trait that presents similarities with a given disorder is a good endophenotype [34]. The endophenotype approach emphasizes the punctuality of model building—the fact that a good model should represent singular phenomena with a high degree of selectivity and is different from the phenomenon being modeled [35].

The search for endophenotypes is not straightforward, since there are no a priori criteria to determine if a particular element/phenomenon/symptom of a mental disorder reflects the disorder as a whole, or whether its dysfunction reflects the effect of a single gene [11]. Proposed endophenotypes range from clinical characterizations, to neurophysiological and neuropsychological measures, to “structural measures of specific, functionally important regions of the brain” [11, p. 704]. Endophenotypes do not necessarily “have to capture specific symptoms that are a part of the clinical diagnosis, but rather may focus on a core process or function that is abnormal in the clinical population under study and that is thought to be related to the manifestation of the illness” [6, p. 883]. As a result, the endophenotype approach is best understood as a strategy to select dependent variables that must show a hypothesized pattern of outcomes.

In the prototypical article on the endophenotype approach, the focus on reductive strategies is justified on the need to pander to behavioral genetics [33]; it is very unlikely that candidate genes can be identified which, when dysfunctional, produce the whole array of symptoms from a given disorder. It’s not surprising, then, that the criteria used to validate an endophenotype are related to genetics:

1. “The endophenotype is associated with illness in the population.
2. The endophenotype is heritable.
3. The endophenotype is primarily state-independent (manifests in an individual whether or not illness is active).
4. Within families, endophenotype and illness co-segregate. (…)
5. The endophenotype found in affected family members is found in nonaffected family members at a higher rate than in the general population” [33, p. 639]

Since endophenotypes represent more defined measures that, it is argued, involve fewer genes, fewer interacting levels, and activation of a single set of neuronal circuits [34], adopting an endophenotype approach could benefit modeling: “we believe that the future development of animal models for psychiatric disorders (not necessarily for the actions of medications) will require a greater focus on validated endophenotypes rather than on symptom-based models” [34, p. 116]. Adopting such an approach could increase the translational value of the model, given that endophenotypes should be derived from human research. For example, while current animal models of bipolar disorder observe behavior or the results of pharmacological manipulation [24, 25], it has been proposed that focusing on different endophenotypes as dependent variables (hyperactivity, irritability, insomnia, aggression, sexual behavior, responsiveness to drugs and reinforcers, reduced concentration, and risk-taking) instead of etiological mechanisms is productive for modeling [23]. These need not correlate with overt phenotype-based models already in existence; in fact, “the current standard of [many] rodent phenotypes to make a high-impact paper is questionable given the nature of the genetics of these disorders” [34, p. 117].

The endophenotype approach is especially relevant in the context of the National Institute of Mental Health (NIMH) Research Domain Criteria (RDoC) system [1]. This framework was proposed 8 years ago to facilitate bridging basic neuroscience research and mental health by introducing an alternative categorization system [18, 19, 39], based on five behavioral domains: (1) positive valence systems, (2) negative valence systems, (3) arousal/regulation systems, (4) systems for social processes, and (5) cognitive systems. It is easy to see how these behavioral domains can represent behavioral endophenotypes, and therefore the RdoC system can be interpreted as supporting “endophenotype-based comparison of animals and humans on an objective neurobiological basis across all behavioural domains” [1, p. 51]. Thus, RDoC is thought to facilitate animal modeling as long as the researcher is able to “assume a model is an endophenotype model […]; [and] assign the experimental endophenotype to 1 of the 5 RDoC domains” [1, p. 52]. Criticism of the first step—assuming the endophenotype approach—can be seen below; one should also note, however, that the RDoC approach has not been met without criticism (e.g., Phillips [55] suggested that the over-emphasizing on reduction led the RDoC approach to view psychiatric disorders as machines whose parts can be studied independently and mechanistically disassembled, which does not appear to be the case).

Although the endophenotype approach certainly increases throughput, an important criticism is that it can lead us to underestimate the importance of interactions between behavioral domains in psychopathology [17, 42, 47, 77]. For example, sleep problems are usually associated with major depressive disorder, and attention deficit hyperactivity disorder has a motor hyperactive component in addition to impulsivity and cognitive components. The use of an endophenotype is unable to dissect a disorder’s specific neurobehavioral domains from comorbidity [42]. Again, *zeitgeist* is as important as other variables here; for example, anxiety and depression symptoms were conflated in all systems from Greek medicine to the rise of biological psychiatry in the nineteenth Century [32]. Moreover, the co-morbidity of anxious and depressive symptoms is high [16], and anxiety and mood disorders share genetic and neurobiological determinants [43]. These observations suggest that a combination of distinct but interacting domains can be mistaken for a clinical endophenotype [48], and therefore a model could benefit from targeting specific domain interplays. Stewart and Kalueff [66] argued that, in addition to the “traditional” types of validity (face, predictive, and construct validity), a good model should also possess inter-relational validity—that is, the ability to target the interplay between various disordered domains.

One problem with this domain interplay approach is that it is time-consuming. A solution is using tests with a wide array of endpoints (dependent variables), allowing the researcher to register as many parameters as possible. The elevated plus-maze, for example, is a test of anxiety that targets several different domains (exploratory behavior, activity, risk assessment); in fact, the use of “ethogram-based” endpoints (e.g., stretched-attend postures, rearing, head dips) can increase the ability of the test to detect serotonergic compounds [56, 75]. Ethogram-based endpoints have been used successfully in zebrafish tests for anxiety-like behavior [14, 51]. Another solution, typically used in the genetics literature, is the use of batteries of specialized tests that focus on different domains [65]; this, however, is time-consuming and requires complex statistical analyses.

An alternative is the use of “hybrid” tests which target different domains [41]. Clear and cross-species examples are the novel object task, which measures both memory and neophobia [10, 26, 59], and the family of holeboard type tests, which simultaneously allow measuring spatial working and reference memory, motivation, exploration, anxiety-related behaviors, and stereotypies in a large range of species [73]. The lizard defense test battery [50] and the rat elevated T-maze [68] both measure anxiety and fear, and the chick separation distress model has endpoints for anxiety and depression-like behavior [76]. These tests are examples of an approach in which a test assesses several different domains simultaneously [41].

This approach can be combined with a “smart battery”, with a block of hybrid tests that exploit the effects of the previous exposure. Kalueff et al. [41, 42] exemplify this with the following battery: an animal is first exposed to the open-field to evaluate baseline anxiety and activity phenotypes, novelty-evoked grooming behavior, within-trial habituation, and potential stereotypies. The animal is then subjected to the acquisition trial of the Morris water maze, and struggling behavior is evaluated in this stage as per Porsolt’s forced swim test. Instead of drying animals and returning them to the homecage, swim-induced grooming behavior and activity levels are registered in an observation cylinder. Finally, the subsequent trials of the Morris water maze can be carried out.

Important criticism on the proposal of using multiple tests and/or a test battery is that

improving test validity and reliability through improved biological understanding may actually obviate the need for multiple tests. By analogy, if one wants to go to the moon, it is probably more sensible to aim one rocket accurately than to send several randomly in the hope that one of them will get there [17, p. 1176].

Of course, this criticism only makes sense when the use of multiple tests is carried out “a-theoretically”, as a “shotgun” approach to try hit a target that is very commonly used in the behavioral phenotyping field. That is not always the case, but Kalueff et al. [41, 42] recognize that hybridizing test conditions can hinder the generalizability and interpretation of results, therefore decreasing external validity, given that “the domains that are being screened may not be discrete at the neurobehavioral levels, and an animal’s reaction to the given ‘hybrid’ test conditions could be different than in any of the single-domain paradigms” (p. 1175). They argue, however, that (in addition to being more cost-effective) the use of hybrid test conditions enables an answer to the domain interplay problem, allowing the researcher to model clinically relevant phenomena (including comorbidity) that are difficult to target in single-domain models. Moreover, the use of hybrid test conditions, the authors argue, enable “a better focus on the newly appreciated ‘continuum’ nature of brain pathogenesis” [41, p. 1176]. Finally, the use of “smart batteries” with fewer but more informative tests is supposed to reduce the impact of stress on subsequent behaviors [41], eliminating potential confounds, and allows to dissociate distinct aspects of a syndrome [17].

Another criticism is that the use of multiple dependent variables—either using hybrid test conditions or test batteries—dramatically reduces power, due to the requirement to use corrections for multiple comparisons, decreasing reliability and, as a consequence, increasing the number of animals needed for discovery instead of the intended reduction [74]. As animal models should be hypothesis based, it may be interesting to explicitly hypothesize the pattern of findings if multiple tests are applied, and to include hypotheses about the relationships between different measures/endpoints. However, most of the time the statistical structure of the relationship between symptoms in the original disorder is unknown [27]. Moreover, the multidimensional nature of the domain interplay approach makes calculation of sample sizes very difficult, due to the multiplicity of statistical models that can be used to define the relationship between variables. Finally, multiple testing may impair the welfare of an animal that is subjected to a battery of tests [74], in particular if these tests have aversive properties and/or harm the animal.

As Kim and colleagues [44] pointed out, tests that can be used in animals and humans are helpful for translating results derived from animal models to humans and to human psychopathological conditions and their treatment. For example, touchscreen-based operant tests have been developed that enable the implementation of tests used in human research and diagnostics in different animal species, in particular rodents (e.g., [13, 44]). The same holds true for cognitive bias tests which have been developed to assess the emotional state of humans and non-human species using a cognitive task [57, 58]. Another recent development are automatic, “home cage” testing systems. These systems are increasingly been used to ‘phenotype’ rodent mutants ([40, 61, 69). Examples are the “phenotyper” [21, 22], a trainable computer vision system for capturing mouse behavior in the home-cage environment [40], and the “IntelliCage” [45, 49]. These systems may have a number of advantages, such as among others testing of the animals in the environment in which they live (avoiding confounding effects of transportation to and testing of animals in an unfamiliar environment) and enabling long term observations, lasting days or weeks. Also, they are not prone to observer bias.

In addition to these dependent variables, good models should also be reliable, and therefore confounding variables, such as locomotor effects, need to be taken into account. Willner [78, 79] treated this as an issue of predictive validity, since, e.g., drugs with non-specific locomotor effects can produce false positives in some tests and screens (including the elevated plus-maze and Porsolt’s forced swim test). This discussion falls beyond the scope of the present article, but has been approached fully elsewhere [72].

The nature of the analogy discussed so far has focused mainly on dependent variables—that is, what is (are) the appropriate endpoint(s) to study. What differentiates a test from a model, however, is not the strategy used to select a dependent variable, but whether the independent variable—that is, the manipulation that is used to induce the disorder—is valid. This is the issue of validity that has been extensively discussed elsewhere (e.g., [8, 66, 70, 78, 79]), but an important issue is that of construct validity—the relationship between the best theory available regarding the target disorder and the model. Construct validity usually refers to theories about etiology and pathogenesis [8], but can include other aspects that are related to the disorder, such as pharmacological isomorphism and ethological aspects [52]. A full treatment of construct validity falls beyond the scope of this article; however, a relevant theoretical framework in experimental psychopathology is the diathesis–stress approach.

## The diathesis–stress approach as a framework for producing animal models

The diathesis–stress approach is a neurobehavioral and psychological theory that attempts to explain mental disorders as the result of an interplay between predispositional (biological and/or psychological) vulnerabilities (diathesis) and stressful life-events [38]. Thus, this framework is useful to explore how biological (genetic or developmental) traits interact with environmental stressors (or protective factors) to produce (or avoid) disorders.

Belzung and Lemoine [8] proposed a general framework for producing animal models that can be useful to understand the difference between models and tests. In this framework (see Figure 1 in Belzung and Lemoine [8]), the analogy is not just between organisms, but between the processes by which both the non-human animal and humans develop the disorder. In that sense, an initial organism consists of a set of mechanisms that is defined by genetic properties; it can be a normal animal or an animal with a naturally occurring deficit. This organism is then exposed to etiological factors; recognizing the role of development on psychopathology, they proposed that early environmental factors transform the initial organism into a vulnerable organism. “The initial organism can be either vulnerable or non-vulnerable from a genetic point of view. Therefore some models aim directly at the transformation of an initial, vulnerable organism into a pathological organism; however on most models this defines the second step” [8]. Moreover, following the differential susceptibility theory [7], positive influences (such as environmental enrichment) should also increase the level of functioning, either avoiding the transformation of the vulnerable to a pathological organism, or increasing functioning in initial organisms that were not exposed to early environmental factors or triggering factors.

Triggering factors occurring in adulthood can transform the vulnerable organism into a pathological organism, equivalent to van der Staay’s concept of “animal with deficits” [70, 71]. The differences between the pathological organism and the initial organism define the neurobehavioral mechanisms underlying the disorder. The effects of this manipulation, therefore, are assessed as the dependent variables of interest—behavioral symptoms and biological markers that can be assessed at the level of the symptom [8], the endophenotype [34], the domain [41]), or multiple domains [42].

An interesting example of this reasoning is found in a recent paper on zebrafish developmental psychopathology [80]. Animals in the early larval stage (roughly equivalent to in utero and newborn mammals) were exposed to either dexamethasone (a glucocorticoid receptor agonist), or to an antisense glucocorticoid receptor morpholino for 5 days (the critical period for the development of the stress axis), thus mimicking increased or decreased stress. Both treatments decreased cortisol levels in the embryos, but only dexamethasone-treated embryos showed decreased anxiety-like behavior [80]. The authors did not use a single test, but rather assessed behavior in the novel tank test, open field, and novel object tests. Moreover, dexamethasone increased the expression of the glucocorticoid receptor in interrenal cells, while the morpholino decreased the expression of the mineralocorticoid receptor in the brain. Consistently, dexamethasone-treated animals showed normal basal cortisol levels, but increased cortisol after stress [80]. Thus, the authors used an early environmental factor to produce a vulnerable organism, and assessed behavior and biological markers for this organism, in an interesting application of the diathesis–stress approach. This animal model is thus the result of a set of operations (independent variables, forming a vulnerable organism) that produce effects on dependent variables (behavioral endpoints and biological markers). Importantly, the zebrafish is not the animal model (do not confuse animal model with model organism), nor are the behavioral tests used to assess the effect.

In a similar approach, still using zebrafish [3, 4], exposed zebrafish larvae to ethanol, mimicking Fetal Alcohol Spectrum Disorder. They showed that this early environmental factor decreased thigmotaxis in the open-field test, scototaxis (preference for dark environments) in the light/dark test, and geotaxis in the novel tank test in adults [4]. Basal cortisol levels were not altered in adults, and ethanol-exposed animals showed blunted cortisol responses to a stressor [3]. Both cases underline the idea that, in order to model a disorder, one needs minimally well-formed causal models of the disorder in humans so that these causal relationships can be modeled.

## Conclusions

The theory-ladenness of models suggest that not only are they dependent on the current scientific *zeitgeist*, but also that they are deeply dependent on the quality of our theories regarding psychopathology *as well as* our theories and understanding of animal behavior. This, of course, creates a central position for an interdisciplinary approach in the process of model building, with psychologists, neuroscientists, ethologists, laboratory animal scientists, and pharmacologists contributing to concatenate data and theory from different fields and produce an essentially behavioral theory that can be translated more easily to the simulation.

The use of behavioral models, as a field of behavioral neuroscience, has been the province of geneticists and pharmacologists, given that these scientists are most directly interested in the applications of models; however, other behavioral scientists (psychologists, ethologists, behavioral neuroscientists, etc.) play an important role in increasing construct validity and, as a consequence, the translational value of a given model. This transdisciplinary approach, “addressing a common problem against the background of a shared conceptual framework by employing theories, concepts and scientific methods of the different disciplines involved” [71], can help further define issues of validity, epistemic uses, and semantic issues. Future refinements of this proposition are expected to attract more researchers in the behavioral sciences to the field, which is in dire need of them.

## Abbreviations

DNA: deoxyribonucleic acid
CAM: causal analog model
HAM: hypothetical analog model
NIMH: National Institute of Mental Health
RdoC: Research Domain Criteria

## Footnotes

1 There are many different senses of the term “model” that can bring confusion. For example, the diathesis-stress theory is normally called “diathesis-stress model”, and “model” here has a very different meaning—that of a theoretical model. In an attempt to dispel the confusion, we reserve the term “model” to refer to behavioral models.
